# Laboratory and Instrumental Risk Factors Associated with a Sudden Cardiac Death Prone ECG Pattern in the General Population: Data from the Brisighella Heart Study

**DOI:** 10.3390/jcm10040640

**Published:** 2021-02-08

**Authors:** Pierangelo Coppola, Arrigo Francesco Giusepp Cicero, Federica Fogacci, Sergio D’Addato, Stefano Bacchelli, Claudio Borghi, on behalf of the Brisighella Heart Study Group

**Affiliations:** 1 Hypertension and Atherosclerosis Research Group, Medical and Surgical Sciences Department, Sant’Orsola-Malpighi University Hospital, Via Albertoni 15, 40138 Bologna, Italy; pierangelocoppola@gmail.com (P.C.); federicafogacci@gmail.com (F.F.); sergio.daddato@unibo.it (S.D.); stefano.bacchelli@aosp.bo.it (S.B.); claudio.borghi@unibo.it (C.B.); 2 IRCCS Azienda Ospedaliero-Universitaria di Bologna, S. Orsola-Malpighi University Hospital, 40138 Bologna, Italy

**Keywords:** sudden cardiac death, pulse-wave velocity, serum uric acid, NSAIDs

## Abstract

Sudden cardiac death (SCD) remains a daunting problem and a major public health issue. We applied the validated Electrocardiogram (ECG) score to the Brisighella Heart Study (BHS) cohort, in order to verify if there were also other recognized laboratory and instrumental risk factors for cardiovascular disease associated with a sudden death risk-prone pattern. We examined the ECG traces of 1377 participants of the 2016 BHS survey and identified 33 subjects at high risk for SCD (while 1344 subjects had no cumulative ECG abnormalities). Serum uric acid (SUA) and carotid-femoral pulse wave velocity (cfPWV) values were significantly higher in the high-risk cohort (*p* < 0.05) and were both independently associated with the presence of ECG abnormalities [Odd ratio (OR) = 2.14, *p* < 0.05–OR = 1.23, *p* < 0.05, respectively]. A similar independent correlation was found with long-term non-steroid anti-inflammatory drugs (NSAIDs) use, more widespread among high-risk subjects (OR = 1.19, *p* < 0.05). Conversely, the analysis did not show any significant association with impaired renal function (*p* = 0.09). This study showed that long-term NSAID use and high SUA and cfPWV values are independent risk factors for ECG abnormalities predictive of SCD. These findings herald the need for further prospective research to identify the optimal combination of SCD risk markers in order to prevent fatal events.

## 1. Introduction

Sudden cardiac death (SCD) is defined as a natural death due to cardiac causes, heralded by abrupt loss of consciousness within 1 h of the onset of acute symptoms; pre-existing heart disease may have been known to be present, but the time and mode of death are unexpected [1]. Conversely, approximately 50% of all deaths occur in patients without a prior diagnosis of heart disease who did not meet low Left Ventricular Ejection Fraction (LVEF) criteria, which occupies a central position in current guidelines for prophylactic Implantable Cardioverter-Defibrillators (ICD) implantation [2]. Therefore, some combinations of arrhythmic risk markers beyond LVEF have been proposed and the recent Finnish Electrocardiogram (ECG) score [3], based on specific ECG alterations, successfully identifies general population subjects with a high SCD risk. This study set out to elucidate the relationship between the presence of the ECG abnormalities associated with progressively increasing risk for SCD and several other clinical parameters in order to provide new potential risk stratification tools.

In this context, we applied the ECG score to the Brisighella Heart Study (BHS) cohort, in order to check if some other laboratory and instrumental risk factors for cardiovascular disease are also associated with a SCD risk-prone pattern.

## 2. Patients and Methods

### 2.1. The Brisighella Heart Study

The BHS is a longitudinal population model active since 1972 on a randomized sample, which is representative of the population of the rural North-Italian village of Brisighella. The complete study protocol has been previously described elsewhere [4]. Briefly, participants were clinically evaluated at baseline and every 4 years thereafter, by collecting a large set of clinical data and biochemical parameters. Mortality and morbidity data, as well as the incidence of the main cardiovascular risk factors, were recorded throughout the entire study. The study protocol has been approved by the Institutional Ethical Board of the University Hospital of Bologna (Code: BrixFollow-up_1972-2024) and it has been performed in accordance with the ethical standards laid down in the 1964 Declaration of Helsinki and its later amendments. All involved subjects signed an informed consent form prior to their inclusion in the study.

### 2.2. The 2016 Brisighella Heart Study Survey

During the 2016 BHS Survey, we consecutively interviewed 1538 subjects about their personal and family history (with specific attention to lifestyle and dietary habits, smoking status, and pharmacological treatment) and recorded a physical examination (including anthropometric data), resting blood pressure (BP) and heart rate, the results of a standard 12-lead electrocardiogram and fasting biochemical analyses. Waist circumference (WC) was measured as the narrowest body diameter between the arcus costarum and the crista iliaca. Height was evaluated with the person standing erect, with bare-feet together and eyes directed straight ahead. Weight was measured twice, and the average of these two measures was used. Body mass index (BMI) was calculated as weight in kilograms divided by height in meters squared (kg/m^2^). Systolic (SBP) and diastolic blood pressure (DBP) were measured three times at 1-min intervals with a standard sphygmomanometer and with the subject in the seated position and after 5 min of quiet rest. The average value of the three measurements was taken as a study variable [5]. Biochemical analyses were carried out on venous blood from the basilic vein. Subjects were fasted for at least 12 h at the time of sampling. All available routine laboratory parameters were analyzed with standardized methods by trained personnel [6], evaluating fasting plasma glucose (FPG), total cholesterol (TC), triglycerides (TG), high-density lipoprotein cholesterol (HDL-C), low-density lipoprotein cholesterol (LDL-C), apolipoprotein AI (apoAI), apolipoprotein B-100 (apoB-100), lipoprotein(a) (Lp(a)), aspartate aminotransferase (ALT), alanine aminotransferase (AST), gamma-glutamyl-transferase (GGT), total bilirubin (TB), creatinine, estimated glomerular filtration rate (eGFR), SUA, and creatinine phosphokinase (CPK). Furthermore, carotid-femoral pulse wave velocity (cfPWV) was noninvasively measured by the Vicorder^®^ apparatus (Skidmore Medical Ltd., Bristol, UK) which is a validated, commercially available, operator-independent device that determines brachial oscillometric BP using a cuff placed around the upper arm. All the measurements were recorded with the subject in the supine resting position. Brachial pressure waveforms were recorded with the same cuff using a volume displacement technique. PWV was determined as the ratio of pulse travel distance to pulse transit time derived from 2-point diastolic pulse wave analysis. Pulse transit time was determined from the foot-to-foot real-time shift between simultaneous 2-point-recorded pulse wave curves using an in-built cross-correlation algorithm based on the peak of the second derivative of the pressure curve. Pulse waves were recorded upon automatic cuff inflation to approximately 60 mmHg over at least 10 pulsations. Furthermore, central systolic and diastolic BP and augmentation index were assessed applying device-specific brachial pulse wave analysis. All travel distances were measured separately for each assessment using a flexible tape. Travel distance for cfPWV was measured directly from the suprasternal notch to the center of the femoral cuff. Central BP parameters were derived from brachial BP waveforms self-calibrated to brachial SBP and brachial DBP, according to a previously described brachial-to-aortic transfer function [7]. Vicorder apparatus was already used in other epidemiological studies, as well [8,9].

### 2.3. ECG Risk Score

Resting ECG traces were recorded from all subjects at a speed of 25 mm/s. After excluding missing or unreadable ECG traces, 1405 ECGs were manually analyzed. We excluded subjects with atrial fibrillation, atrial flutter, left or right bundle branch block, II/III degree atrioventricular block, ventricular pre-excitation, a pacemaker rhythm, or rare ECG findings not representative of the general population (*n* = 21). We also excluded subjects with missing data (*n* = 7). Then, a cumulative ECG risk score was applied to the remaining 1377 ECGs, in order to identify subjects with a high SCD risk. This score specifically predicts SCD risk relying on the presence of ≥3 specific ECG alterations: heart rate > 80 beats per minute (bpm), PR interval > 220 ms, QRS duration > 110 ms, left ventricular hypertrophy, and T wave inversion [3]. The score has recently been developed by a Finnish research group who demonstrated as among 6830 Mini-Finland Health Survey participants the risk for SCD progressively increased with each additional ECG abnormality, whereby subjects with ≥3 ECG abnormalities exhibited the highest risk [Hazard Ratio (HR) = 10.23; 95% CI 5.29–19.80; *p* < 0.001] for SCD. This association persisted in the complete follow-up of about 20 years in the multivariate model and subsists in both subjects with or without a CAD or heart failure diagnosis, with no statistically significant effect modifications. Similar results were achieved among the 10,617 subjects of the score validation cohort [3].

### 2.4. Statistical Analysis

A full descriptive analysis was carried out for the considered variables. A chi-squared test was performed for all the qualitative parameters while all the quantitative variables were compared by Student’s *t*-test. Non-normally distributed parameters were then log-transformed before going on with the analyses. The analyses were repeated by sex. A multiple logistic regression analysis was finally carried out in order to identify the main predictors of ECG proarrhythmic alterations in the high-risk subgroup identified by the ECG risk score. All tests were carried out using SPSS 25.0 for Windows (IBM Corporation, Armonk, NY, USA). A significance level of 0.05 was considered valid for every test.

## 3. Results

### 3.1. High-Risk Subjects Identification

**Figure 1:**
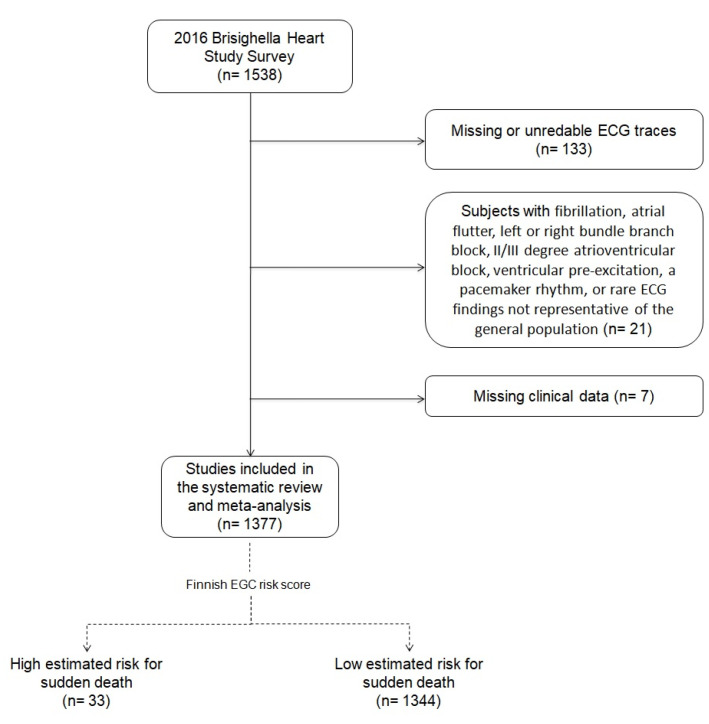
Flow-chart resuming the selection criteria applied to the full cohort to select the investigated subjects.

Among the analyzed 1377 resting ECG traces, 33 (2.4%) turned out to have the variables associated with increased mortality and were hence considered at high risk for SCD, according to the Finnish EGC risk score (Figure 1). The mean age of low-risk subjects was 57.5 ± 15.6 years old, whereas those at high risk were 70.7 ± 13.7 years old. The prevalence of high-risk female patients was 51.5%.

### 3.2. High-Risk Subjects’ History

Categorical variables analyzed referred to pharmacological and familial anamnestic information gained during our interviews. Among these, long-term NSAIDs [both the cyclooxygenase inhibitors (COX-1 and COX-2)] and antihypertensive drug use showed a statistically significant different frequency distribution between the high-risk and low-risk subgroups. In particular, as many as 7.4% of low-risk subjects regularly take NSAIDs, while 29% of the high-risk ones have this habit (*p* < 0.001) (Table 1). Furthermore, up to 63.6% of high-risk subjects take antihypertensive drugs daily (*p* < 0.001) (Table 2), however, the main classes of antihypertensive drugs used (renin-angiotensin-aldosterone inhibitors, calcium-channel blockers, beta-blockers, and diuretics) were equally distributed between groups. The 17.8% of low-risk subjects and 31.3% of high-risk subjects assumed statins.

In contrast, no statistically significant difference was found comparing the following anamnestic variables between the two cohorts: lipid-lowering drugs use, obstructive sleep apnea syndrome (OSAS), snoring, smoking habit, personal history of major cardiac events (MACE), and family history of diabetes, dyslipidemias, hypertension, coronary artery disease and cardiovascular disease (Supplementary Tables S1–S10).

### 3.3. High-Risk Subjects’ Anthropometric Data

Table 3 shows the results obtained from the anthropometric measurements of our population and its relative probability distribution parameters.

Student’s *t*-test showed as an index of central obesity (ICO) and neck, hip, and waist circumference mean values were statistically different between the two cohorts. Particularly they all had higher values in the high-risk subgroup (Table 3).

### 3.4. High-Risk Subjects’ Hemodynamic Parameters

In order to assess potential hemodynamic common phenotypes among the high-risk subjects, several parameters concerning blood pressure and the arterial stiffness of the circulatory system were measured noninvasively in both cohorts as showed in Table 4.

The hypothesis tests applied to our data point out that SBP, DBP, and mean arterial blood pressure (MAP) values, just like the ones for aortic blood pressure, were all higher in the high-risk subgroup with a statistically significant difference compared to the low subgroup subjects (*p* < 0.05) (Table 4). Analogously, cfPWV mean values were lower in the cohort at low risk with a similar significant difference (*p* < 0.05) (Table 4).

### 3.5. High-Risk Subjects’ Laboratory Parameters

Several biochemical analyses were carried out on venous blood samples measuring routine laboratory parameters concerning hepatic, lipid, and glucose metabolism as well as kidney function. The results of our measurements are shown in Table 5.

The analysis of these data also showed some differences comparing the results obtained from our measurements between the two cohorts. Particularly SUA concentration, FPG, creatinine, apoA1, and lipid accumulation product (LAP) mean values were all significantly higher in the group at high risk for SCD. Conversely, eGFR values were significantly lower in the same group, regardless of the formula used. No statistical differences were highlighted from the application of the same tests to the other biochemical variables (Table 5).

Since all the measured parameters had a strong interdependence, in order to assess the actual causal relationship between the data we gathered and the cumulative presence of the pro-arrhythmic ECG alterations mentioned above, we carried out a multiple logistic regression eliminating any confounding factors. The results showed that long-term NSAIDs use and higher values of SUA concentration and cfPWV were independently and significantly able to predict subjects’ categorical placement in the high-risk subgroup.

Specifically, the strongest relation was observed with SUA concentration. In fact, the higher values of SUA the more increased probability to belong with the high-risk cohort [Odd Ratio (OR) = 2.14; 95% Confidence Interval (CI) 1.38–3.33; *p* < 0.05]. Similar association, albeit with slightly lower statistical significance, was found with the cfPWV values (OR = 1.23; CI 1.01–1.49; *p* < 0.05) and the reported long-term NSAIDs use (OR = 1.19; 95% CI 1.04–1.87; *p* < 0.05). The remaining parameters evaluated, including those referred to the kidney function, have not shown any significant causal relation. (Supplementary Table S11).

Repeating the analysis by sex, we did not identify any specific sex-related difference between the risk groups.

From 2016 to 2000, only one subject died because of SCD (correctly identified by the risk score as a high-risk patient), while 9 subjects died because of other diseases.

## 4. Discussion

Nowadays the challenge is still on to predict SCD. Primary prevention strategies, aimed at implanting ICDs in patients with reduced LVEF, are still inadequate so that SCD is even now a major global health issue [10]. The research of a scoring system able to identify subjects at high risk for SCD has been occupying a leading role in the last decades, and the recently validated Finnish ECG risk score [3] has shown promising results. However, risk stratification may continue to improve by combining pro-arrhythmic ECG abnormalities and other known SCD risk markers in new predictive models. Available information about such kinds of relationships is still limited and inconsistent. Our study broadens the knowledge about this topic by analyzing the distribution of several cardiovascular risk factors in a high SCD risk cohort, as defined by the presence of cumulative ECG abnormalities.

According to our findings, plasma lipids and smoking habit were not associated with a higher risk score. As for lipids, most of the subjects with hypercholesterolemia were treated with statins, which could reduce the incidence of ischemic ECG changes regardless of lipid concentrations, as shown in previous studies [11]. Furthermore, LDL-C mainly predicts ECG signs of previous myocardial infarction [12], which is just one of the five ECG alterations included in the Finnish Score. In fact, the ECG risk score includes also ECG depolarization abnormalities whose underlying mechanism could be led back more to cardiac remodeling than to atherosclerosis disease.

With regard to the cigarette smoking habit, as with cholesterol, it showed to be able to predict ECG signs of previous myocardial infarction [12], which is again one of the five ECG alterations included in the Finnish Score. In fact, it could be considered as a possible predictor of ventricular arrhythmogenesis only in as much as it has been associated with a prolonged Tp-e interval, increased Tp-e/QT ratio, and Tp-e/QTc ratio [13], but these variables are not included in the Finnish score. Indeed, since smoking accelerates atrioventricular nodal conduction [14], it is less likely that smokers meet the other ECG Finnish risk score criteria, such as QRS > 150 ms.

Despite that the literature suggests that sex could influence some ECG parameters [15], we did not observe any sex-related difference between risk groups in our cohort. This could probably be related to the relatively small dimension of the high-risk group.

Firstly, we demonstrated that long-term NSAIDs use is related to a high SCD risk. This might potentially be explained by the known NSAIDs’ mechanism of inhibition of vasodilating mediators production, such as prostaglandins, resulting in a pro-thrombotic state induction with a persistent condition of hydro saline retention and tendency to higher blood pressure values [16]. The detected ECG alterations might hence be regarded as the organ damage expression due to the afore-mentioned pathological processes, in agreement with results of previous studies [17].

Secondly, to the best of our knowledge, this is the first cross-sectional study that found an association between the gold standard measurement of arterial stiffness-namely cfPWV- and SCD. These results confirm what previous studies demonstrated about the association between high cfPWV values and ECG repolarization abnormalities [18]. This might be explained by the electrophysiological cardiac remodeling consequent to the increased ventricular afterload elicited by arterial stiffness. Otherwise, the association might be based on subendocardial ischaemias due to coronary microvascular damage, which is promoted by high arterial stiffness [19].

Finally, from our results, SUA concentration as well seemed to be a good predictor of electrical myocardium instability. Several evidence already demonstrated that higher SUA levels are associated with ECG abnormalities such as LVH [20], Q-waves [12], conduction defects [21], T-wave inversion [22], ventricular arrhythmia onset [23], and our data are in line with these results. Underlying mechanisms could be led back to coronary endothelial dysfunction [24] or cardiac remodeling [25], both due to hyperuricemia. Furthermore, we observed that this association is independent of the glomerular filtration rate. Therefore, the relation between ECG alterations and hyperuricemia cannot be considered as an impaired kidney function epiphenomenon (Table S2) but a strong independent association itself.

Our study has some limitations that should be acknowledged. The survey enrolled individuals from BHS who belong to a culturally and dietary homogeneous community that may not be fully representative of the general population. In fact, the mean intake of proteins and animal saturated fats is quite elevated in this area due to the local food culture, despite some educational intervention on the general population [26,27]. Then, the sample size of the high-risk subgroup is relatively small, though representative of the full cohort, and consequently did not allow sub-analyses by age classes or other subgroups. Furthermore, our cardiovascular evaluation was based mainly on classical risk factors and ECG pattern, while no specific cardiac parameter (e.g., troponin or NTproBNP) was available. Finally, since only one subject died because of SCD in the following 4 years, we were not able to build survival curves.

Of course, the Finnish ECG score could be affected by antiarrhythmic drug use. However, by excluding from the analysis subjects with known heart rhythm disorders, we also excluded subjects taking antiarrhythmic drugs.

## 5. Conclusions

In conclusion, long-term NSAIDs use, high cfPWV and SUA concentration values are independent risk factors for pro-arrhythmic ECG alterations onset. Further efforts are needed in order to combine these markers in predictive models and consequently help to improve SCD risk stratification.

## Figures and Tables

**Table 1:** Contingency table about non-steroid anti-inflammatory drugs (NSAIDs) use in the 2016 Brisighella Heart Study (BHS) survey subpopulations, according to the ECG risk score.

			Long-Term NSAIDs Use	
			No	Yes
Estimated risk of sudden death	Low	Number of subjects	1189	95
		% within risk of sudden death	92.6%	7.4%
		% in long-term NSAIDs use	98.2%	91.3%
		% of the total	90.4%	7.2%
	High	Number of subjects	22	9
		% within risk of sudden death	71%	29.0%
		% in Long-term NSAIDs use	1.8%	8.7%
		% of the total	1.7%	0.7%
Whole sample		Number of subjects	1211	104
		% within risk of sudden death	92.1%	7.9%
		% in long-term NSAIDs use	100%	100%
		% of the total	92.1%	7.9%

**Table 2:** Contingency table about antihypertensive drugs use in the 2016 BHS Survey subpopulations, according to the ECG risk score.

			Estimated Risk of Sudden Death	
			Low	High
Antihypertensive drugs use	No	Number of subjects	909	12
		% within antihypertensive drugs use	98.7%	1.3%
		% within risk of sudden death	67.7%	36.4%
		% of the total	66.1%	0.9%
	Yes	Number of subjects	434	21
		% within antihypertensive drugs use	95.4%	4.6%
		% within risk of sudden death	32.3%	63.6%
		% of the total	31.5%	1.5%
Whole sample		Number of subjects	1343	33
		% within antihypertensive drugs use	97.6%	2.4%
		% within risk of sudden death	100%	100%
		% of the total	97.6%	2.4%

**Table 3:** Descriptive and comparative analysis of anthropometric characteristics of the investigated cohorts.

	Low Estimated Risk for Sudden Death (Mean ± Standard Deviation)	High Estimated Risk for Sudden Death (Mean ± Standard Deviation)	Mean Differences	95% Confidence Interval of the Differences		Sig. (2-Tailed)
				Lower Limit	Upper Limit	
Waist circumference (cm)	92.03 ± 13.22	97.27 ± 8.98	−5.23	−9.85	−0.62	0.026
Hip circumference (cm)	100.88 ± 11.17	103.71 ± 6.92	−2.83	−6.66	1.01	0.148
Body Mass Index (kg/m^2^)	26.68 ± 4.64	27.63 ± 2.84	−0.95	−2.55	0.64	0.241
Waist/Hip ratio	0.91 ± 0.09	0.93 ± 0.08	−0.03	−0.06	0.01	0.109
Index of Central Obesity	0.56 ± 0.08	0.60 ± 0.06	−0.04	−0.07	−0.01	0.005
Wrist circumference (cm)	17.16 ± 1.79	17.38 ± 1.26	−0.22	−0.84	0.39	0.479
Arm circumference (cm)	28.46 ± 3.39	27.91 ± 2.65	0.55	−0.62	1.72	0.356
Neck circumference (cm)	36.91 ± 4.15	38.47 ± 3.43	−1.56	−2.99	−0.13	0.032

**Table 4:** Descriptive and comparative analysis of hemodynamic parameters laboratory parameters of the investigated cohorts.

	Low Estimated Risk for Sudden Death (Mean ± Standard Deviation)	High Estimated Risk for Sudden Death (Mean ± Standard Deviation)	Mean Differences	95% Confidence Interval of the Differences		Sig. (2-Tailed)
				Lower Limit	Upper Limit	
Systolic blood pressure (mmHg)	140.23 ± 20.69	151.10 ± 28.2	−10.87	−18.44	−3.3	0.005
Diastolic blood pressure (mmHg)	73.22 ± 9.53	78.77 ± 15.66	−5.55	−9.07	−2.03	0.002
Pulse pressure (mmHg)	67.01 ± 16.83	72.33 ± 20.73	−5.32	−11.45	0.82	0.089
Aortic blood pressure (mmHg)	137.27 ± 20.82	147.53 ± 27.88	−10.26	−17.88	−2.65	0.008
Aortic pulse pressure (mmHg)	64.06 ± 16.82	68.77 ± 20.76	−4.71	−10.84	1.43	0.133
Augmentation Index (%)	25.62 ± 9.28	24.63 ± 11.27	0.98	−2.40	4.37	0.569
Cardiac Output (L/min)	7.05 ± 2.28	8.09 ± 2.8	−1.04	−1.87	−0.21	0.015
Total Peripheral Resistance (PRU)	0.93 ± 0.29	0.88 ± 0.28	0.05	−0.05	0.16	0.314
Stroke Volume (mL)	112.56 ± 34.88	114.53 ± 36.84	−1.97	−14.64	10.7	0.760
Mean Arterial Pressure (mmHg)	95.56 ± 11.84	102.87 ± 18.25	−7.32	−11.67	−2.96	0.001
Carotid-femoral Pulse Wave Velocity (m/s)	9 ± 2.30	10.23 ± 3.34	−1.3	−2.14	−0.45	0.003
Pulse Pressure Index	0.47 ± 0.06	0.47 ± 0.08	−0.001	−0.024	0.023	0.955
Right Ankle-Brachial Index	1.13 ± 0.16	1.16 ± 0.15	−0.03	−0.1	0.03	0.281
Left Ankle-Brachial Index	1.13 ± 0.16	1.15 ± 0.13	−0.03	−0.09	0.04	0.432
Cardiac Index	4.01 ± 1.46	4.53 ± 2.08	−0.53	−1.64	0.58	0.351

**Table 5:** Descriptive and comparative analysis of laboratory parameters of the investigated cohorts.

	Low Estimated Risk for Sudden Death (Mean ± Standard Deviation)	High Estimated Risk for Sudden Death (Mean ± Standard Deviation)	Mean Differences	95% Confidence Interval of the Differences		Sig. (2-Tailed)
				Lower Limit	Upper Limit	
Total Cholesterol (mg/dL)	217.27 ± 40.63	217.09 ± 39.78	0.18	−13.86	14.22	0.980
Triglycerides (mg/dL)	118.57 ± 68.09	161.03 ± 144.63	−42.46	−66.95	−17.98	0.001
High-Density Lipoprotein Cholesterol (mg/dL)	51.99 ± 15.01	53.21 ± 19.82	−1.22	−6.46	4.01	0.647
Low-Density Lipoprotein Cholesterol (mg/dL)	141.77 ± 36.79	135.92 ± 38.85	5.85	−7.28	18.98	0.382
Serum uric acid (mg/dL)	5.27 ± 1.33	5.86 ± 1.35	−0.59	−1.05	−0.13	0.013
Fasting plasma glucose (mg/dL)	95.18 ± 20.32	98.48 ± 35.19	−3.3	−10.49	3.89	0.368
Apolipoprotein A1 (mg/dL)	154.75 ± 28.51	150.06 ± 28.47	4.69	−5.16	14.55	0.350
Apolipoprotein B-100 (mg/dL)	91.96 ± 20.58	89.3 ± 21.24	2.65	−4.47	9.77	0.465
Creatinine (mg/dL)	1.03 ± 0.19	1.15 ± 0.38	−0.12	−0.19	−0.05	0.001
Aspartate aminotransferase (U/L)	23.48 ± 17.91	24.27 ± 8.11	−0.8	−6.93	5.34	0.799
Alanine aminotransferase (U/L)	24.55 ± 20.2	22.06 ± 7.72	2.49	−4.42	9.4	0.480
Creatinine Phosphokinase (U/L)	130.02 ± 146.85	119.82 ± 63.42	10.2	−40.08	60.48	0.691
Gamma-Glutamyl-Transferase (U/L)	26.8 ± 26.34	32.03 ± 40.76	−5.23	−14.48	4.03	0.268
Low-Density Lipoprotein Cholesterol/Apolipoprotein B-100	1.56 ± 0.32	1.53 ± 0.26	0.03	−0.08	0.15	0.576
High-Density Lipoprotein Cholesterol/Apolipoprotein A1	0.33 ± 0.07	0.35 ± 0.08	−0.01	−0.04	0.01	0.241
Lipoprotein(a) (mg/dL)	22.72 ± 30.06	20.84 ± 29.22	1.88	−8.5	12.27	0.722
Visceral Adiposity Index	3.27 ± 1.67	3.8 ± 1.33	−0.55	−1.5	0.41	0.259
eGFR-CKD Epi (mL/min)	71.32 ± 15.52	62.65 ± 17.56	8.68	3.3	14.06	0.002
eGFR-MDRD (mL/min)	70.20 ± 13.81	65.12 ± 16.79	5.08	0.28	9.89	0.038
eGFR-Cockcroft Gault (mL/min)	75.24 ± 24.1	62.58 ± 25.75	12.66	4.31	21	0.003
eGFR-Mayo Quadratic Formula (mL/min)	89.92 ± 17.3	79.76 ± 22.37	10.16	4.13	16.19	0.001
Lipid Accumulation Product	44.4 ± 36.49	65.23 ± 58.6	−20.82	−33.86	−7.79	0.002
Hepatic Steatosis Index	37.8 ± 5.83	37.48 ± 3.84	0.32	−1.69	2.32	0.754

CKD = Chronic kidney disease; eGFR = Estimated glomerular filtration rate.

## Data Availability

Data supporting findings of this analysis are available from the University of Bologna. Data are available from the authors with the permission of the University of Bologna.

## Supplementary Materials

The following are available online at https://www.mdpi.com/2077-0383/10/4/640/s1, Tables S1–S10: Contingency tables about other variables analyzed in the 2016 BHS Survey subpopulations, according to the ECG risk score; Table S11: Excluded variables in multiple regression.

## References

[1] Priori S.G., Aliot E., Blomstrom-Lundqvist C., Bossaert L., Breithardt G., Brugada P., Camm A.J., Cappato R., Cobbe S.M., Di Mario C. (2001). Task Force on Sudden Cardiac Death of the European Society of Cardiology. Eur. Heart J..

[2] Chugh S.S., Reinier K., Teodorescu C., Evanado A., Kehr E., Al Samara M., Mariani R., Gunson K., Jui J. (2008). Epidemiology of Sudden Cardiac Death: Clinical and Research Implications. Prog. Cardiovasc. Dis..

[3] Holkeri A., Eranti A., Haukilahti M.A.E., Kerola T., Kenttä T.V., Tikkanen J.T., Anttonen O., Noponen K., Seppänen T., Rissanen H. (2020). Predicting sudden cardiac death in a general population using an electrocardiographic risk score. Heart.

[4] Cicero A.F., D’Addato S., Santi F., Ferroni A., Borghi C., Brisighella Heart Study (2012). Leisure-time physical activity and cardiovascular disease mortality: The Brisighella Heart Study. J. Cardiovasc. Med..

[5] Cicero A.F.G., Fogacci F., Giovannini M., Grandi E., D’Addato S., Borghi C., for the Brisighella Heart Study group (2019). Interaction between low-density lipoprotein-cholesterolaemia, serum uric level and incident hypertension. J. Hypertens..

[6] Cicero A.F.G., Rosticci M., Fogacci F., Grandi E., D’Addato S., Borghi C. (2017). High serum uric acid is associated to poorly controlled blood pressure and higher arterial stiffness in hypertensive subjects. Eur. J. Intern. Med..

[7] Hickson S., Butlin M., Broad J., Avolio A.P., Wilkinson I.B., McEniery C.M. (2009). Validity and repeatability of the Vicorder apparatus: A comparison with the SphygmoCor device. Hypertens. Res..

[8] Parsons T.J., Sartini C., Ellins E.A., Halcox J.P.J., Smith K.E., Ash S., Lennon L.T., Wannamethee S.G., Lee I.M., Whincup P.H. (2016). Objectively measured physical activity, sedentary time and subclinical vascular disease: Cross-sectional study in older British men. Prev. Med..

[9] Müller J., Ewert P., Hager A. (2015). Increased aortic blood pressure augmentation in patients with congenital heart defects e a crosssectional study in 1125 patients and 322 controls. Int. J. Cardiol..

[10] Kong M.H., Fonarow G.C., Peterson E.D., Curtis A.B., Hernandez A.F., Sanders G.D., Thomas K.L., Hayes D.L., Al-Khatib S.M. (2011). Systematic review of the incidence of sudden cardiac death in the United States. J. Am. Coll. Cardiol..

[11] Shimizu M., Koizumi J., Miyamoto S., Origasa H., Mabuchi H., HOLICOS-PAT Study Group (2005). Electrocardiographic events and cholesterol reduction with pravastatin in patients with hypercholesterolemia: The Hokuriku Lipid Coronary Heart Disease Study-Pravastatin Atherosclerosis Trial. Int. J. Cardiol..

[12] Cicero A.F., Rosticci M., Tocci G., Bacchelli S., Urso R., D’Addato S., Borghi C. (2015). Serum uric acid and other short-term predictors of electrocardiographic alterations in the Brisighella Heart Study cohort. Eur. J. Intern. Med..

[13] İlgenli T.F., Tokatlı A., Akpınar O., Kılıçaslan F. (2015). The Effects of Cigarette Smoking on the Tp-e Interval, Tp-e/QT Ratio and Tp-e/QTc Ratio. Adv. Clin. Exp. Med..

[14] Irfan A.B., Arab C., DeFilippis A.P., Lorkiewicz P., Keith R.J., Xie Z., Bhatnagar A., Carll A.P. (2021). Smoking Accelerates Atrioventricular Conduction in Humans Concordant with Increased Dopamine Release. Cardiovasc. Toxicol..

[15] Guo X., Li Z., Liu Y., Yu S., Yang H., Zheng L., Zhang Y., Sun Y. (2016). Sex-specific association between serum uric acid and prolonged corrected QT interval: Result from a general rural Chinese population. Medicine.

[16] Schjerning A.M., McGettigan P., Gislason G. (2020). Cardiovascular effects and safety of (non-aspirin) NSAIDs. Nat. Rev. Cardiol..

[17] Bindu S., Mazumder S., Bandyopadhyay U. (2020). Non-steroidal anti-inflammatory drugs (NSAIDs) and organ damage: A current perspective. Biochem. Pharmacol..

[18] Mozos I. (2015). The link between ventricular repolarization variables and arterial function. J. Electrocard..

[19] Cooper L.L., Palmisano J.N., Benjamin E.J. (2016). Microvascular function contributes to the relation between aortic stiffness and cardiovascular events: The Framingham Heart Study. Circ. Cardiovasc. Imaging.

[20] Cicero A.F., Rosticci M., Reggi A., Derosa G., Parini A., Grandi E., D’Addato S., Borghi C. (2015). Relationship Between Serum Uric Acid and Electrocardiographic Alterations in a Large Sample of General Population: Data from the Brisighella Heart Study. High Blood. Press. Cardiovasc. Prev..

[21] Mantovani A., Rigolon R., Pichiri I., Morani G., Bonapace S., Dugo C., Zoppini G., Bonora E., Targher G. (2017). Relation of elevated serum uric acid levels to first-degree heart block and other cardiac conduction defects in hospitalized patients with type 2 diabetes. J. Diabetes Complicat..

[22] Schwartz I.F., Grupper A., Chernichovski T., Grupper A., Hillel O., Engel A., Schwartz D. (2011). Hyperuricemia attenuates aortic nitric oxide generation, through inhibition of arginine transport, in rats. J. Vasc. Res..

[23] Yamada S., Suzuki H., Kamioka M., Kamiyama Y., Saitoh S., Takeishi Y. (2012). Uric acid increases the incidence of ventricular arrhythmia in patients with left ventricular hypertrophy. Fukushima J. Med. Sci..

[24] Silbernagel G., Hoffmann M.M., Grammer T.B., Boehm B.O., März W. (2013). Uric acid is predictive of cardiovascular mortality and sudden cardiac death in subjects referred for coronary angiography. Nutr. Metab. Cardiovasc. Dis..

[25] Iwashima Y., Horio T., Kamide K., Rakugi H., Ogihara T., Kawano Y. (2006). Uric acid, left ventricular mass index, and risk of cardiovascular disease in essential hypertension. Hypertension.

[26] Cicero A., Fogacci F., Grandi E., Rizzoli E., Bove M., D’Addato S., Borghi C. (2020). Prevalent Seasoning and Cooking Fats, Arterial Stiffness and Blood Lipid Pattern in a Rural Population Sample: Data from the Brisighella Heart Study. Nutrients.

[27] Cicero A., Fogacci F., Desideri G., Grandi E., Rizzoli E., D’Addato S., Borghi C. (2019). Arterial Stiffness, Sugar-Sweetened Beverages and Fruits Intake in a Rural Population Sample: Data from the Brisighella Heart Study. Nutrients.

