# Supplementary material for: Laboratory and Instrumental Risk Factors Associated with a Sudden Cardiac Death Prone ECG Pattern in the General Population: Data from the Brisighella Heart Study
Source: J Clin Med. 2021 Feb 8;10(4):640. doi: 10.3390/jcm10040640 (PMC7914833; doi:10.3390/jcm10040640)
Supplement: Supplementary file 1 [file jcm-10-00640-s001.pdf]

J. Clin. Med

Supplementary

			Sudden Death Risk		
			Low	High	Total
**Smoking habit**	**No**	**Count**	749a	19a	768
		**% within Smoking habit**	97,5%	2,5%	100,0%
		**% within Sudden Death Risk**	56,1%	57,6%	56,1%
		**% of the total**	54,8%	1,4%	56,1%
	**Current**	**Count**	339a	10a	349
		**% within Smoking habit**	97,1%	2,9%	100,0%
		**% within Sudden Death Risk**	25,4%	30,3%	25,5%
		**% of the total**	24,8%	0,7%	25,5%
	**Former**	**Count**	247a	4a	251
		**% within Smoking habit**	98,4%	1,6%	100,0%
		**% within Sudden Death Risk**	18,5%	12,1%	18,3%
		**% of the total**	18,1%	0,3%	18,3%
**Total**		**Count**	1335	33	1368
		**% within Smoking habit**	97,6%	2,4%	100,0%
		**% within Sudden Death Risk**	100,0%	100,0%	100,0%
		**% of the total**	97,6%	2,4%	100,0%

Table S1. Contingency table about smoking habit in the Brisighella Heart Study Survey (2016) subpopulations, according to the ECG risk score.

			Sudden Death Risk		
			Low	High	Total
Snoring	Yes	Count	559a	13a	572
		% within Snoring	97,7%	2,3%	100,0%
		% within Sudden Death Risk	41,6%	39,4%	41,6%
		% of the total	40,6%	0,9%	41,6%
	No	Count	536a	16a	552
		% within Snoring	97,1%	2,9%	100,0%
		% within Sudden Death Risk	39,9%	48,5%	40,1%
		% of the total	39,0%	1,2%	40,1%
	Don't know	Count	248a	4a	252
		% within Snoring	98,4%	1,6%	100,0%
		% within Sudden Death Risk	18,5%	12,1%	18,3%
		% of the total	18,0%	0,3%	18,3%
Total		Count	1343	33	1376
		% within Snoring	97,6%	2,4%	100,0%
		% within Sudden Death Risk	100,0%	100,0%	100,0%
		% of the total	97,6%	2,4%	100,0%

Table S2. Contingency table about snoring in the Brisighella Heart Study Survey (2016) subpopulations, according to the ECG risk score.

			Sudden Death Risk		
			Low	High	Total
Sleep apnoea	Yes	Count	967a	26a	993
		% within Sleep apnoea	97,4%	2,6%	100,0%
		% within Sudden Death Risk	74,4%	81,3%	74,6%
		% of the total	72,7%	2,0%	74,6%
	No	Count	83a	1a	84
		% within Sleep apnoea	98,8%	1,2%	100,0%
		% in Sudden Death Risk	6,4%	3,1%	6,3%
		% of the total	6,2%	0,1%	6,3%
	Don't know	Count	249a	5a	254
		% within Sleep apnoea	98,0%	2,0%	100,0%
		% in Sudden Death Risk	19,2%	15,6%	19,1%
		% of the total	18,7%	0,4%	19,1%
Total		Count	1299	32	1331
		% within Sleep apnoea	97,6%	2,4%	100,0%
		% in Sudden Death Risk	100,0%	100,0%	100,0%
		% of the total	97,6%	2,4%	100,0%

Table S3. Contingency table about sleep apnoea in the Brisighella Heart Study Survey (2016) subpopulations, according to the ECG risk score.

			Sudden Death Risk		
			Low	High	Total
MACE_TOT	No	Count	**1260a**	**29a**	**1289**
		% in MACE_TOT	**97,8%**	**2,2%**	**100,0%**
		% within Sudden Death Risk	**93,8%**	**87,9%**	**93,7%**
		% of the del total	**91,6%**	**2,1%**	**93,7%**

	Yes	Count	83a	4a	87
		% in MACE_TOT	95,4%	4,6%	100,0%
		% within Sudden Death Risk	6,2%	12,1%	6,3%
		% of the del total	6,0%	0,3%	6,3%
Total		Count	1343	33	1376
		% in MACE_TOT	97,6%	2,4%	100,0%
		% within Sudden Death Risk	100,0%	100,0%	100,0%
		% of the del total	97,6%	2,4%	100,0%

Table S4. Contingency table about Total Major Adverse Cardiovascular Events (MACE_TOT) in the Brisighella Heart Study Survey (2016) subpopulations, according to the ECG risk score.

			Sudden Death Risk		
			Low	High	Total
ANTIHLP	No	Count	1064a	22a	1086
		% within ANTIHLP	98,0%	2,0%	100,0%
		% within Sudden Death Risk	82,2%	68,8%	81,8%
		% of the total	80,2%	1,7%	81,8%
	Yes	Count	231a	10a	241
		% within ANTIHLP	95,9%	4,1%	100,0%
		% within Sudden Death Risk	17,8%	31,3%	18,2%
		% of the total	17,4%	0,8%	18,2%
Total		Count	1295	32	1327
		% within ANTIHLP	97,6%	2,4%	100,0%
		% within Sudden Death Risk	100,0%	100,0%	100,0%
		% of the total	97,6%	2,4%	100,0%

Table S5. Contingency table about hypolipidemic drugs use (ANTIHLP) in the Brisighella Heart Study Survey (2016) subpopulations, according to the ECG risk score.

			Sudden Death Risk		
			Low	High	Total
FAMHPT	No	Count	625a	18a	643
		% within FAMHPT	97,2%	2,8%	100,0%
		% within Sudden Death Risk	46,5%	54,5%	46,7%
		% of the total	45,4%	1,3%	46,7%
	Yes	Count	718a	15a	733
		% within FAMHPT	98,0%	2,0%	100,0%
		% within Sudden Death Risk	53,5%	45,5%	53,3%
		% of the total	52,2%	1,1%	53,3%
Total		Count	1343	33	1376
		% within FAMHPT	97,6%	2,4%	100,0%
		% within Sudden Death Risk	100,0%	100,0%	100,0%
		% of the total	97,6%	2,4%	100,0%

Table S6. Contingency table about familiarity with hypertension (FAMHPT) in the Brisighella Heart Study Survey (2016) subpopulations, according to the ECG risk score.

			Sudden Death Risk		
			Low	High	Total
FAMHLP	No	Count	750a	24a	774
		% within FAMHLP	96,9%	3,1%	100,0%
		% within Sudden Death Risk	56,9%	72,7%	57,3%
		% of the total	55,6%	1,8%	57,3%
	1	Count	567a	9a	576
		% within FAMHLP	98,4%	1,6%	100,0%
		% within Sudden Death Risk	43,1%	27,3%	42,7%
		% of the total	42,0%	0,7%	42,7%

Total	Count	1317	33	1350
	% within FAMHLP	97,6%	2,4%	100,0%
	% within Sudden Death Risk	100,0%	100,0%	100,0%
	% of the total	97,6%	2,4%	100,0%

Table S7. Contingency table about familiarity with dyslipidemias (FAMHLP) in the Brisighella Heart Study Survey (2016) subpopulations, according to the ECG risk score.

			Sudden Death Risk		
			Low	High	Total
FAMDM	No	Count	987a	29a	1016
		% within FAMDM	97,1%	2,9%	100,0%
		% within Sudden Death Risk	73,5%	87,9%	73,8%
		% of the total	71,7%	2,1%	73,8%
	Yes	Count	356a	4a	360
		% within FAMDM	98,9%	1,1%	100,0%
		% within Sudden Death Risk	26,5%	12,1%	26,2%
		% of the total	25,9%	0,3%	26,2%
Total		Count	1343	33	1376
		% within FAMDM	97,6%	2,4%	100,0%
		% within Sudden Death Risk	100,0%	100,0%	100,0%
		% of the total	97,6%	2,4%	100,0%

Table S8. Contingency table about familiarity with Diabetes (FAMDM) in the Brisighella Heart Study Survey (2016) subpopulations, according to the ECG risk score.

			Sudden Death Risk		
			Low	High	Total
FAMCAD	No	Count	975a	24a	999

		% within FAMCAD	97,6%	2,4%	100,0%
		% in Sudden Death Risk	72,6%	72,7%	72,6%
		% of the total	70,9%	1,7%	72,6%
	Yes	Count	368a	9a	377
		% within FAMCAD	97,6%	2,4%	100,0%
		% in Sudden Death Risk	27,4%	27,3%	27,4%
		% of the total	26,7%	0,7%	27,4%
Total		Count	1343	33	1376
		% within FAMCAD	97,6%	2,4%	100,0%
		% in Sudden Death Risk	100,0%	100,0%	100,0%
		% of the total	97,6%	2,4%	100,0%

Table S9. **Contingency table about familiarity with Coronary Artery Disease (FAMCAD) in the Brisighella Heart Study Survey (2016) subpopulations, according to the ECG risk score.**

			Sudden Death Risk		
			Low	High	Total
FAMCVD	No	Count	1093a	29a	1122
		% within FAMCVD	97,4%	2,6%	100,0%
		% within Sudden Death Risk	81,4%	87,9%	81,5%
		% of the total	79,4%	2,1%	81,5%
	Yes	Count	250a	4a	254
		% within FAMCVD	98,4%	1,6%	100,0%
		% within Sudden Death Risk	18,6%	12,1%	18,5%
		% of the total	18,2%	0,3%	18,5%
Total		Count	1343	33	1376
		% within FAMCVD	97,6%	2,4%	100,0%
		% within Sudden Death Risk	100,0%	100,0%	100,0%
		% of the total	97,6%	2,4%	100,0%

Table S10. **Contingency table about familiarity with Cardiovascular Disease (FAMCVD) in the Brisighella Heart Study Survey (2016) subpopulations, according to the ECG risk score.**

			Point	df	Sign.
**Phase 1**	**Variables**	**Age (years)**	4,900	1	,027
		**Smoking habit**	,976	2	,614
		**Smoking habit(1)**	,923	1	,337
		**Smoking habit(2)**	,701	1	,402
		**Snoring**	,865	2	,649
		**Snoring(1)**	,382	1	,536
		**Snoring(2)**	,008	1	,929
		**Sleep apnoea**	1,679	2	,432
		**Sleep apnoea(1)**	1,572	1	,210
		**Sleep apnoea(2)**	,726	1	,394
		**MACE_TOT_12(1)**	1,466	1	,226
		**ANTIHPT_12(1)**	1,717	1	,190
		**ANTIHLP_12(1)**	1,093	1	,296
		**ANTIDM_12(1)**	,239	1	,625
		**FANS_12(1)**	7,471	1	,006
		**FAMHPT_12(1)**	,370	1	,543
		**FAMHLP_12(1)**	,868	1	,352
		**FAMDM_12(1)**	,546	1	,460
		**FAMCAD_12(1)**	,026	1	,872
		**FAMCVD_12(1)**	1,297	1	,255
		**Waist circumference (cm)**	,029	1	,864
		**Body Mass Index (kg/m2)**	,457	1	,499
		**Heart rate (bpm)**	4,288	1	,038
		**SBP (mmHg)**	1,497	1	,221
		**DBP (mmHg)**	2,867	1	,090
		**PP (mmHg)**	,353	1	,552
		**Aortic BP (mmHg)**	1,288	1	,256

		**Aortic PP (mmHg)**	,240	1	,624
		**Augmentation Index**	2,905	1	,088
		**Cardiac Output**	1,569	1	,210
		**Stroke Volume**	,026	1	,871
		**cfPWV (m/s)**	5,600	1	,018
		**TG (mg/dL)**	,017	1	,898
		**HDL-C (mg/dL)**	,342	1	,559
		**LDL-C (mg/dL)**	,420	1	,517
		**FPG (mg/dL)**	,275	1	,600
		**ApoB (mg/dL)**	,025	1	,874
		**AST_12**	,016	1	,899
		**ALT_12**	,146	1	,702
		**CPK_12**	,005	1	,942
		**GGT_12**	,009	1	,925
		**Lp(a) (mg/dL)**	,189	1	,664
		**VAI**	,106	1	,745
		**eGFR-CKD Epi (ml/min)**	3,982	1	,046
		**SUACR**	2,004	1	,157
**Phase 2**	**Variables**	**Age (years)**	2,881	1	,090
		**Smoking habit**	,749	2	,688
		**Smoking habit(1)**	,634	1	,426
		**Smoking habit(2)**	,669	1	,413
		**Snoring**	,621	2	,733
		**Snoring(1)**	,256	1	,613
		**Snoring(2)**	,007	1	,933
		**Sleep apnoea**	1,302	2	,521
		**Sleep apnoea(1)**	1,171	1	,279
		**Sleep apnoea(2)**	,668	1	,414

	**MACE_TOT_12(1)**	,963	1	,326
	**ANTIHPT_12(1)**	1,076	1	,300
	**ANTIHLP_12(1)**	,325	1	,569
	**ANTIDM_12(1)**	,012	1	,913
	**FAMHPT_12(1)**	,634	1	,426
	**FAMHLP_12(1)**	,583	1	,445
	**FAMDM_12(1)**	,230	1	,631
	**FAMCAD_12(1)**	,023	1	,880
	**FAMCVD_12(1)**	,874	1	,350
	**Waist circumference (cm)**	,030	1	,862
	**Body Mass Index (kg/m2)**	,402	1	,526
	**Hear rate (bpm)**	5,028	1	,025
	**SBP (mmHg)**	,741	1	,389
	**DBP (mmHg)**	3,026	1	,082
	**PP (mmHg)**	,029	1	,865
	**Aortic BP (mmHg)**	,576	1	,448
	**Aortic PP (mmHg)**	,002	1	,961
	**Augmentation Index**	4,867	1	,027
	**Cardiac Output**	,740	1	,390
	**Stroke Volume**	,054	1	,816
	**cfPWV (m/s)**	3,891	1	,049
	**TG (mg/dL)**	,039	1	,843
	**HDL-C (mg/dL)**	,156	1	,693
	**LDL-C (mg/dL)**	,137	1	,711
	**FPG (mg/dL)**	,076	1	,783
	**ApoB (mg/dL)**	,001	1	,976
	**AST_12**	,011	1	,917
	**ALT_12**	,137	1	,711

		**CPK_12**	,000	1	,993
		**GGT_12**	,011	1	,918
		**Lp(a) (mg/dL)**	,344	1	,558
		**VAI**	,030	1	,862
		**eGFR-CKD Epi (ml/min)**	2,880	1	,090
		**SUACR**	1,835	1	,175
**Phase 3**	**Variables**	**Age (years)**	3,858	1	,050
		**Smoking habit**	,748	2	,688
		**Smoking habit(1)**	,678	1	,410
		**Smoking habit(2)**	,600	1	,439
		**Snoring**	,508	2	,776
		**Snoring(1)**	,104	1	,747
		**Snoring(2)**	,054	1	,817
		**Sleep apnoea**	1,900	2	,387
		**Sleep apnoea(1)**	1,618	1	,203
		**Sleep apnoea(2)**	1,242	1	,265
		**MACE_TOT_12(1)**	2,399	1	,121
		**ANTIHPT_12(1)**	1,645	1	,200
		**ANTIHLP_12(1)**	,349	1	,555
		**ANTIDM_12(1)**	,067	1	,796
		**FAMHPT_12(1)**	,559	1	,455
		**FAMHLP_12(1)**	,655	1	,418
		**FAMDM_12(1)**	,306	1	,580
		**FAMCAD_12(1)**	,122	1	,727
		**FAMCVD_12(1)**	,961	1	,327
		**Waist circumference (cm)**	,133	1	,715
		**Body Mass Index (kg/m2)**	,540	1	,462
		**SBP (mmHg)**	,907	1	,341

		**DBP (mmHg)**	2,024	1	,155
		**PP (mmHg)**	,179	1	,672
		**Aortic BP (mmHg)**	,833	1	,361
		**Aortic PP (mmHg)**	,137	1	,712
		**Augmentation Index**	3,798	1	,051
		**Cardiac Output**	,283	1	,595
		**Stroke Volume**	,193	1	,660
		**cfPWV (m/s)**	4,597	1	,032
		**TG (mg/dL)**	,016	1	,899
		**HDL-C (mg/dL)**	,099	1	,753
		**LDL-C (mg/dL)**	,354	1	,552
		**FPG (mg/dL)**	,021	1	,885
		**ApoB (mg/dL)**	,076	1	,783
		**AST_12**	,007	1	,931
		**ALT_12**	,289	1	,591
		**CPK_12**	,020	1	,888
		**GGT_12**	,011	1	,917
		**Lp(a) (mg/dL)**	,221	1	,639
		**VAI**	,018	1	,892
		**eGFR-CKD Epi (ml/min)**	3,806	1	,051
		**SUACR**	2,015	1	,156
**Phase 4**	**Variables**	**Age (years)**	2,127	1	,145
		**Smoking habit**	1,040	2	,595
		**Smoking habit(1)**	,778	1	,378
		**Smoking habit(2)**	1,008	1	,315
		**Snoring**	,283	2	,868
		**Snoring(1)**	,004	1	,952
		**Snoring(2)**	,105	1	,746

	**Sleep apnoea**	1,410	2	,494
	**Sleep apnoea(1)**	1,081	1	,298
	**Sleep apnoea(2)**	1,034	1	,309
	**MACE_TOT_12(1)**	2,487	1	,115
	**ANTIHPT_12(1)**	1,163	1	,281
	**ANTIHLP_12(1)**	,052	1	,820
	**ANTIDM_12(1)**	,047	1	,828
	**FAMHPT_12(1)**	,748	1	,387
	**FAMHLP_12(1)**	,235	1	,628
	**FAMDM_12(1)**	,130	1	,718
	**FAMCAD_12(1)**	,093	1	,760
	**FAMCVD_12(1)**	,858	1	,354
	**Waist circumference (cm)**	,010	1	,919
	**Body Mass Index (kg/m2)**	,201	1	,654
	**SBP (mmHg)**	,062	1	,803
	**DBP (mmHg)**	1,215	1	,270
	**PP (mmHg)**	,072	1	,789
	**Aortic BP (mmHg)**	,038	1	,846
	**Aortic PP (mmHg)**	,120	1	,729
	**Augmentation Index**	5,044	1	,025
	**Cardiac Output**	,023	1	,879
	**Stroke Volume**	,044	1	,833
	**TG (mg/dL)**	,011	1	,918
	**HDL-C (mg/dL)**	,081	1	,776
	**LDL-C (mg/dL)**	,326	1	,568
	**FPG (mg/dL)**	,027	1	,869
	**ApoB (mg/dL)**	,135	1	,713
	**AST_12**	,022	1	,883

	**ALT_12**	,223	1	,637
	**CPK_12**	,053	1	,819
	**GGT_12**	,010	1	,919
	**Lp(a) (mg/dL)**	,227	1	,634
	**VAI**	,029	1	,864
	**eGFR-CKD Epi (ml/min)**	2,930	1	,087
	**SUACR**	2,008	1	,156

Table S11. Excluded variables in multiple regression.
